# 5-Fluoro-3-(3-fluoro­phenyl­sulfon­yl)-2-methyl-1-benzofuran

**DOI:** 10.1107/S1600536812031972

**Published:** 2012-07-18

**Authors:** Hong Dae Choi, Pil Ja Seo, Uk Lee

**Affiliations:** a Department of Chemistry, Dongeui University, San 24 Kaya-dong, Busanjin-gu, Busan 614-714, Republic of Korea; b Department of Chemistry, Pukyong National University, 599-1 Daeyeon 3-dong, Nam-gu, Busan 608-737, Republic of Korea

## Abstract

In the title compound, C_15_H_10_F_2_O_3_S, the 3-fluoro­phenyl ring makes a dihedral angle of 79.51 (6)° with the mean plane [r.m.s. deviation = 0.006 (2) Å] of the benzofuran fragment. In the crystal, mol­ecules are linked by weak C—H⋯F and C—H⋯O hydrogen bonds. The crystal structure also exhibits slipped π–π inter­actions between the benzene and furan rings of neighbouring mol­ecules [centroid–centroid distances = 3.563 (3) and 3.820 (3) Å and slippages of 0.358 (3)and 1.551 (3) Å]. In the 3-fluoro­phenyl ring, the F atom is disordered over two positions with site-occupancy factors of 0.887 (3) and 0.113 (3).

## Related literature

For background information and the crystal structures of related compounds, see: Choi *et al.* (2010 ▶, 2012 ▶).
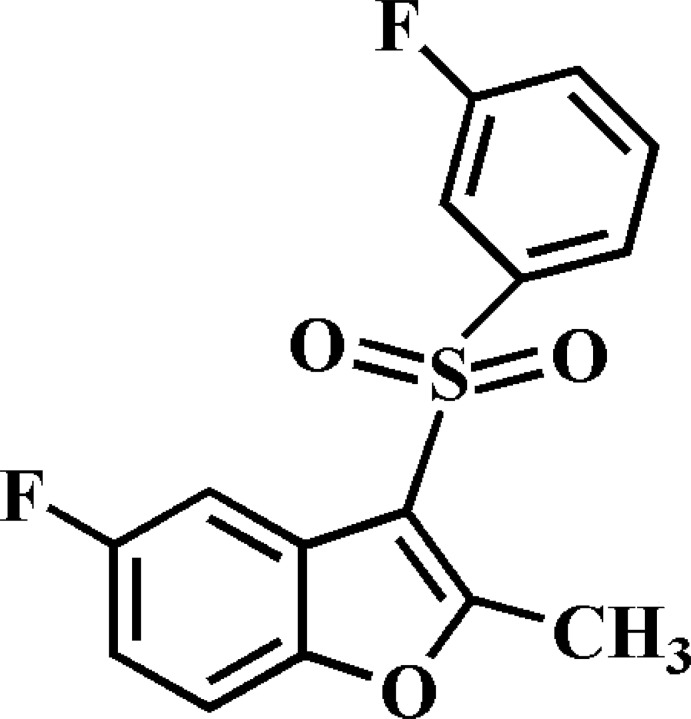


## Experimental

### 

#### Crystal data

C_15_H_10_F_2_O_3_S
*M* *_r_* = 308.29
Monoclinic, 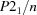

*a* = 7.2772 (2) Å
*b* = 11.0972 (3) Å
*c* = 16.5625 (5) Å
β = 100.698 (2)°
*V* = 1314.28 (6) Å^3^
*Z* = 4
Mo *K*α radiation
μ = 0.28 mm^−1^
*T* = 296 K
0.30 × 0.22 × 0.20 mm

#### Data collection

Bruker SMART APEXII CCD diffractometer
Absorption correction: multi-scan (*SADABS*; Bruker, 2009 ▶) *T*_min_ = 0.663, *T*_max_ = 0.746
12792 measured reflections
3275 independent reflections
2732 reflections with *I* > 2σ(*I*)
*R*_int_ = 0.032

#### Refinement

*R*[*F* ^2^ > 2σ(*F* ^2^)] = 0.037
*wR*(*F* ^2^) = 0.104
*S* = 1.04
3275 reflections
202 parameters
14 restraints
H-atom parameters constrained
Δρ_max_ = 0.36 e Å^−3^
Δρ_min_ = −0.31 e Å^−3^

### 

Data collection: *APEX2* (Bruker, 2009 ▶); cell refinement: *SAINT* (Bruker, 2009 ▶); data reduction: *SAINT*; program(s) used to solve structure: *SHELXS97* (Sheldrick, 2008 ▶); program(s) used to refine structure: *SHELXL97* (Sheldrick, 2008 ▶); molecular graphics: *ORTEP-3* (Farrugia, 1997 ▶) and *DIAMOND* (Brandenburg, 1998 ▶); software used to prepare material for publication: *SHELXL97*.

## Supplementary Material

Crystal structure: contains datablock(s) global, I. DOI: 10.1107/S1600536812031972/pk2432sup1.cif

Structure factors: contains datablock(s) I. DOI: 10.1107/S1600536812031972/pk2432Isup2.hkl

Supplementary material file. DOI: 10.1107/S1600536812031972/pk2432Isup3.cml

Additional supplementary materials:  crystallographic information; 3D view; checkCIF report

## Figures and Tables

**Table 1:** Hydrogen-bond geometry (Å, °)

*D*—H⋯*A*	*D*—H	H⋯*A*	*D*⋯*A*	*D*—H⋯*A*
C5—H5⋯F2^i^	0.93	2.45	3.302 (2)	153
C15—H15⋯O3^ii^	0.93	2.47	3.3754 (19)	165

Symmetry codes: (i) 
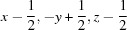
; (ii) 
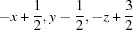
.

**Fig. 1.**
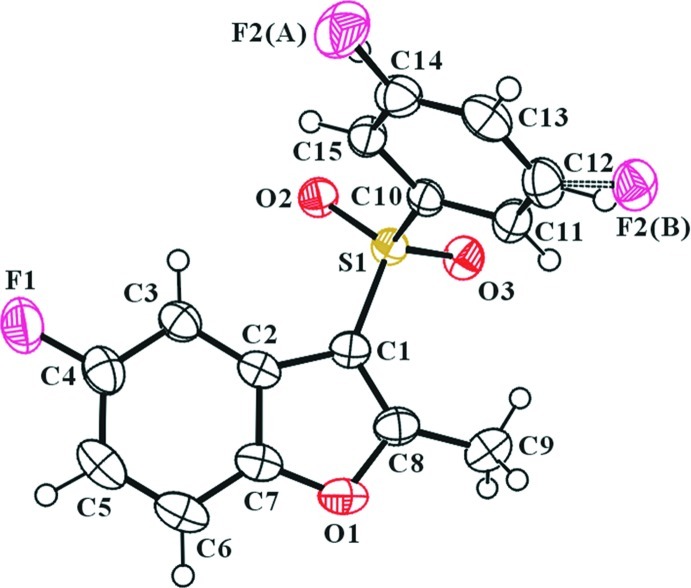
The molecular structure of the title compound with displacement ellipsoids drawn at the 50% probability level. H atoms are presented as small spheres of arbitrary radius. The F atom of the 3-fluorophenyl ring is disordered over two positions with refined site occupancy factors, of 0.887 (3) (part A) and 0.113 (3) (part B).

**Fig. 2.**
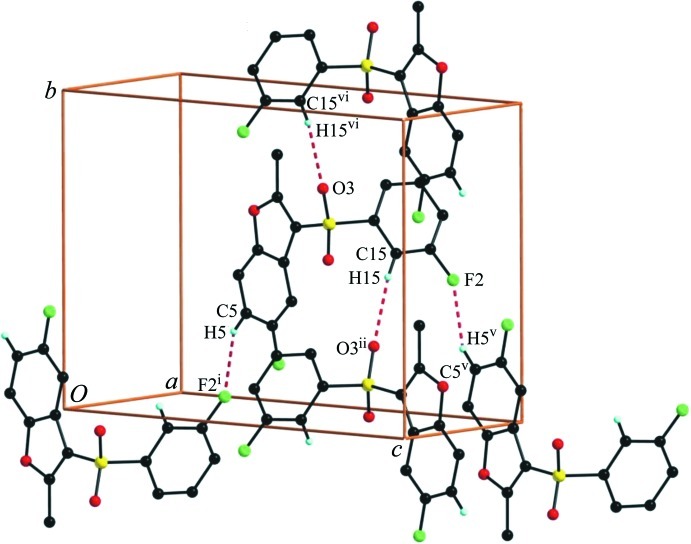
A view of the C–H···F and C–H···O interactions (dotted lines) in the crystal structure of the title compound. H atoms not participating in hydrogen-bonding were omitted for clarity. [Symmetry codes: (i) x - 1/2, - y + 1/2, z - 1/2; (ii) - x + 1/2, y - 1/2, - z + 3/2; (v) x + 1/2, - y + 1/2, z + 1/2; (vi) - x + 1/2, y + 1/2, - z + 3/2.]

**Fig. 3.**
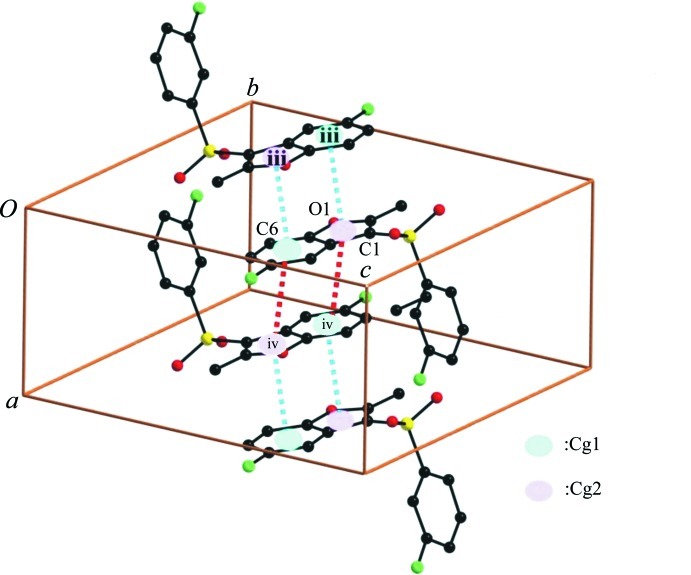
A view of the π–π interactions (dotted lines) in the crystal structure of the title compound. All H atoms were omitted for clarity. [Symmetry codes: (iii) - x, - y + 1, - z + 1; (iv) - x + 1, - y + 1, - z + 1]

## supplementary crystallographic information

### Comment

As a part of our continuing study of 5-fluoro-2-methyl-1-benzofuran derivatives containing 3-(4-fluorophenylsulfonyl) (Choi *et al.*, 2010) and 3-(4-methylphenylsulfonyl) (Choi *et al.*, 2012) substituents, we report herein the crystal structure of the title compound.

In the title molecule (Fig. 1), the benzofuran unit is essentially planar, with a mean deviation of 0.006 (2) Å from the least-squares plane defined by the nine constituent atoms. In the 3-fluorophenyl ring, the F atom is disordered over two positions with site-occupancy factors, from refinement of 0.887 (3) (part A) and 0.113 (3) (part B). The dihedral angle between the 3-fluorophenyl ring and the mean plane of the benzofuran ring is 79.51 (6)°. In the crystal structure (Fig. 2), molecules are connected by weak intermolecular C–H···F and C–H···O hydrogen bonds (Table 1). The crystal packing (Fig. 3) also exhibits slipped π–π interactions between the benzene and furan rings of neighbouring molecules, with Cg1···Cg2^iii^ and Cg1···Cg2^iv^ distances of 3.563 (3) Å & 3.820 (3) Å, and interplanar distances of 3.545 (3) Å & 3.491 (3) Å resulting in slippages of 0.358 (3) Å & 1.551 (3) Å (Cg1 and Cg2 are the centroids of the C2-C7 benzene ring and the C1/C2/C7/O1/C8 furan ring, respectively).

### Experimental

3-Chloroperoxybenzoic acid (77%, 515 mg, 2.3 mmol) was added in small portions to a stirred solution of 5-fluoro-3-(3-fluorophenylsulfanyl)-2-methyl-1-benzofuran (304 mg, 1.1 mmol) in dichloromethane (50 mL) at 273 K. After being stirred at room temperature for 10h, the mixture was washed with saturated sodium bicarbonate solution and the organic layer was separated, dried over magnesium sulfate, filtered and concentrated at reduced pressure. The residue was purified by column chromatography (benzene) to afford the title compound as a colorless solid [yield 73%, m.p. 443-444 K; *R*_f_ = 0.66 (benzene)]. Single crystals suitable for X-ray diffraction were prepared by slow evaporation of a solution of the title compound in ethyl acetate at room temperature.

### Refinement

All H atoms were positioned geometrically and refined using a riding model, with C–H = 0.93 Å for aryl and 0.96 Å for methyl H atoms. *U*_iso_(H) = 1.2*U*_eq_(C) for aryl and 1.5*U*_eq_(C) for methyl H atoms. The positions of methyl hydrogens were optimized rotationally. The F2 atom of the 3-fluorophenyl ring is disordered over two positions with site occupancy factors, from refinement of 0.887 (3) (part A) and 0.113 (3) (part B). The distance of equivalent C-F pairs were restrained to 1.330 (5) Å using the SHELXL-97 command DFIX, and displacement ellipsoids of F2 set were restrained using the SHELXL-97 command ISOR.

### Crystal data

**Table:**

C_15_H_10_F_2_O_3_S	*F*(000) = 632
*M**_r_* = 308.29	*D*_x_ = 1.558 Mg m^−^^3^
Monoclinic, *P*2_1_/*n*	Mo *K*α radiation, λ = 0.71073 Å
Hall symbol: -P 2yn	Cell parameters from 3933 reflections
*a* = 7.2772 (2) Å	θ = 2.2–28.2°
*b* = 11.0972 (3) Å	µ = 0.28 mm^−^^1^
*c* = 16.5625 (5) Å	*T* = 296 K
β = 100.698 (2)°	Block, colourless
*V* = 1314.28 (6) Å^3^	0.30 × 0.22 × 0.20 mm
*Z* = 4	

### Data collection

**Table:**

Bruker SMART APEXII CCD diffractometer	3275 independent reflections
Radiation source: rotating anode	2732 reflections with *I* > 2σ(*I*)
Graphite multilayer monochromator	*R*_int_ = 0.032
Detector resolution: 10.0 pixels mm^-1^	θ_max_ = 28.3°, θ_min_ = 2.2°
φ and ω scans	*h* = −9→9
Absorption correction: multi-scan (*SADABS*; Bruker, 2009)	*k* = −14→14
*T*_min_ = 0.663, *T*_max_ = 0.746	*l* = −22→22
12792 measured reflections	

### Refinement

**Table:**

Refinement on *F*^2^	Secondary atom site location: difference Fourier map
Least-squares matrix: full	Hydrogen site location: difference Fourier map
*R*[*F*^2^ > 2σ(*F*^2^)] = 0.037	H-atom parameters constrained
*wR*(*F*^2^) = 0.104	*w* = 1/[σ^2^(*F*_o_^2^) + (0.0548*P*)^2^ + 0.3108*P*] where *P* = (*F*_o_^2^ + 2*F*_c_^2^)/3
*S* = 1.04	(Δ/σ)_max_ = 0.001
3275 reflections	Δρ_max_ = 0.36 e Å^−^^3^
202 parameters	Δρ_min_ = −0.31 e Å^−^^3^
14 restraints	Extinction correction: *SHELXL97* (Sheldrick, 2008), Fc^*^=kFc[1+0.001xFc^2^λ^3^/sin(2θ)]^-1/4^
Primary atom site location: structure-invariant direct methods	Extinction coefficient: 0.0088 (14)

### Special details

**Table:**

Geometry. All esds (except the esd in the dihedral angle between two l.s. planes) are estimated using the full covariance matrix. The cell esds are taken into account individually in the estimation of esds in distances, angles and torsion angles; correlations between esds in cell parameters are only used when they are defined by crystal symmetry. An approximate (isotropic) treatment of cell esds is used for estimating esds involving l.s. planes.
Refinement. Refinement of F^2^ against ALL reflections. The weighted R-factor wR and goodness of fit S are based on F^2^, conventional R-factors R are based on F, with F set to zero for negative F^2^. The threshold expression of F^2^ > 2sigma(F^2^) is used only for calculating R-factors(gt) etc. and is not relevant to the choice of reflections for refinement. R-factors based on F^2^ are statistically about twice as large as those based on F, and R- factors based on ALL data will be even larger.

### Fractional atomic coordinates and isotropic or equivalent isotropic displacement parameters (Å^2^)

**Table:**

	*x*	*y*	*z*	*U*_iso_*/*U*_eq_	Occ. (<1)
S1	0.21640 (5)	0.63440 (3)	0.70540 (2)	0.03087 (13)	
F1	0.25059 (17)	0.17801 (10)	0.54399 (7)	0.0617 (3)	
O1	0.25258 (16)	0.65312 (11)	0.47256 (7)	0.0437 (3)	
O2	0.13169 (15)	0.52825 (10)	0.73181 (7)	0.0373 (3)	
O3	0.13613 (15)	0.75027 (10)	0.71521 (7)	0.0407 (3)	
C1	0.2307 (2)	0.61487 (14)	0.60279 (9)	0.0324 (3)	
C2	0.24256 (19)	0.49996 (14)	0.56282 (9)	0.0323 (3)	
C3	0.2414 (2)	0.37946 (15)	0.58555 (10)	0.0375 (4)	
H3	0.2332	0.3559	0.6387	0.045*	
C4	0.2532 (2)	0.29710 (17)	0.52459 (11)	0.0437 (4)	
C5	0.2648 (2)	0.32726 (19)	0.44430 (11)	0.0491 (5)	
H5	0.2715	0.2671	0.4059	0.059*	
C6	0.2664 (2)	0.44598 (18)	0.42199 (10)	0.0456 (4)	
H6	0.2745	0.4688	0.3687	0.055*	
C7	0.2553 (2)	0.53018 (16)	0.48228 (9)	0.0369 (4)	
C8	0.2376 (2)	0.70287 (16)	0.54652 (10)	0.0391 (4)	
C9	0.2333 (3)	0.83607 (17)	0.54796 (12)	0.0547 (5)	
H9A	0.2265	0.8632	0.6024	0.082*	
H9B	0.1259	0.8644	0.5101	0.082*	
H9C	0.3449	0.8671	0.5324	0.082*	
C10	0.4518 (2)	0.63802 (13)	0.75603 (9)	0.0298 (3)	
C11	0.5530 (2)	0.74450 (14)	0.75670 (10)	0.0375 (3)	
H11	0.4957	0.8143	0.7332	0.045*	
C12	0.7390 (2)	0.74468 (16)	0.79277 (11)	0.0420 (4)	0.887 (3)
H12	0.8083	0.8150	0.7925	0.050*	0.887 (3)
C12'	0.7390 (2)	0.74468 (16)	0.79277 (11)	0.0420 (4)	0.11
F2'	0.8472 (11)	0.8379 (6)	0.8099 (5)	0.042 (3)	0.113 (3)
C13	0.8244 (2)	0.64277 (16)	0.82925 (10)	0.0409 (4)	
H13	0.9504	0.6434	0.8535	0.049*	
C14	0.7198 (2)	0.54100 (15)	0.82885 (10)	0.0402 (4)	0.887 (3)
F2	0.80371 (19)	0.44337 (11)	0.86633 (8)	0.0619 (4)	0.887 (3)
C14'	0.7198 (2)	0.54100 (15)	0.82885 (10)	0.0402 (4)	0.11
H14'	0.7770	0.4726	0.8546	0.048*	0.113 (3)
C15	0.5338 (2)	0.53388 (14)	0.79236 (9)	0.0354 (3)	
H15	0.4666	0.4626	0.7921	0.042*	

### Atomic displacement parameters (Å^2^)

**Table:**

	*U*^11^	*U*^22^	*U*^33^	*U*^12^	*U*^13^	*U*^23^
S1	0.0314 (2)	0.0312 (2)	0.0326 (2)	0.00158 (14)	0.01275 (15)	−0.00048 (14)
F1	0.0746 (8)	0.0435 (6)	0.0622 (7)	0.0073 (6)	−0.0001 (6)	−0.0119 (5)
O1	0.0432 (7)	0.0576 (8)	0.0303 (6)	−0.0037 (5)	0.0070 (5)	0.0065 (5)
O2	0.0380 (6)	0.0387 (6)	0.0384 (6)	−0.0052 (5)	0.0158 (5)	−0.0007 (5)
O3	0.0392 (6)	0.0379 (6)	0.0478 (7)	0.0076 (5)	0.0157 (5)	−0.0022 (5)
C1	0.0281 (7)	0.0384 (8)	0.0318 (7)	0.0015 (6)	0.0084 (6)	0.0008 (6)
C2	0.0227 (7)	0.0446 (9)	0.0293 (7)	0.0015 (6)	0.0044 (5)	−0.0022 (6)
C3	0.0337 (8)	0.0431 (9)	0.0347 (8)	0.0026 (6)	0.0040 (6)	−0.0033 (6)
C4	0.0373 (9)	0.0446 (10)	0.0459 (9)	0.0041 (7)	−0.0007 (7)	−0.0090 (7)
C5	0.0390 (9)	0.0659 (13)	0.0404 (9)	0.0022 (8)	0.0023 (7)	−0.0205 (9)
C6	0.0346 (8)	0.0720 (13)	0.0296 (8)	−0.0023 (8)	0.0046 (6)	−0.0066 (8)
C7	0.0265 (7)	0.0530 (10)	0.0306 (7)	−0.0012 (6)	0.0044 (6)	0.0004 (7)
C8	0.0342 (8)	0.0475 (10)	0.0360 (8)	0.0008 (7)	0.0072 (6)	0.0050 (7)
C9	0.0671 (13)	0.0459 (11)	0.0512 (11)	−0.0017 (9)	0.0116 (9)	0.0113 (8)
C10	0.0329 (7)	0.0301 (7)	0.0287 (7)	0.0007 (6)	0.0117 (6)	−0.0034 (5)
C11	0.0417 (8)	0.0307 (8)	0.0419 (8)	−0.0004 (6)	0.0126 (7)	0.0003 (6)
C12	0.0406 (9)	0.0416 (9)	0.0457 (9)	−0.0081 (7)	0.0131 (7)	−0.0085 (7)
C12'	0.0406 (9)	0.0416 (9)	0.0457 (9)	−0.0081 (7)	0.0131 (7)	−0.0085 (7)
F2'	0.038 (4)	0.041 (5)	0.048 (5)	−0.014 (3)	0.013 (3)	−0.005 (3)
C13	0.0344 (8)	0.0540 (10)	0.0345 (8)	0.0003 (7)	0.0072 (6)	−0.0090 (7)
C14	0.0445 (9)	0.0409 (9)	0.0348 (8)	0.0089 (7)	0.0065 (7)	0.0014 (7)
F2	0.0601 (8)	0.0469 (8)	0.0715 (9)	0.0124 (6)	−0.0070 (7)	0.0121 (6)
C14'	0.0445 (9)	0.0409 (9)	0.0348 (8)	0.0089 (7)	0.0065 (7)	0.0014 (7)
C15	0.0416 (8)	0.0303 (8)	0.0355 (8)	−0.0009 (6)	0.0103 (6)	−0.0006 (6)

### Geometric parameters (Å, º)

**Table:**

S1—O3	1.4339 (11)	C6—H6	0.9300
S1—O2	1.4349 (11)	C8—C9	1.479 (2)
S1—C1	1.7354 (15)	C9—H9A	0.9600
S1—C10	1.7627 (16)	C9—H9B	0.9600
F1—C4	1.361 (2)	C9—H9C	0.9600
O1—C8	1.366 (2)	C10—C15	1.386 (2)
O1—C7	1.374 (2)	C10—C11	1.391 (2)
C1—C8	1.357 (2)	C11—C12	1.374 (2)
C1—C2	1.447 (2)	C11—H11	0.9300
C2—C3	1.390 (2)	C12—C13	1.375 (2)
C2—C7	1.395 (2)	C12—H12	0.9300
C3—C4	1.376 (2)	C13—C14	1.361 (2)
C3—H3	0.9300	C13—H13	0.9300
C4—C5	1.389 (3)	C14—F2	1.3383 (19)
C5—C6	1.369 (3)	C14—C15	1.378 (2)
C5—H5	0.9300	C15—H15	0.9300
C6—C7	1.381 (2)		
			
O3—S1—O2	119.64 (7)	C1—C8—O1	110.14 (15)
O3—S1—C1	108.82 (7)	C1—C8—C9	134.79 (16)
O2—S1—C1	107.65 (7)	O1—C8—C9	115.07 (15)
O3—S1—C10	107.83 (7)	C8—C9—H9A	109.5
O2—S1—C10	107.96 (7)	C8—C9—H9B	109.5
C1—S1—C10	103.84 (7)	H9A—C9—H9B	109.5
C8—O1—C7	107.34 (12)	C8—C9—H9C	109.5
C8—C1—C2	107.87 (13)	H9A—C9—H9C	109.5
C8—C1—S1	126.82 (13)	H9B—C9—H9C	109.5
C2—C1—S1	125.29 (11)	C15—C10—C11	121.37 (15)
C3—C2—C7	119.67 (15)	C15—C10—S1	119.32 (12)
C3—C2—C1	136.08 (14)	C11—C10—S1	119.29 (12)
C7—C2—C1	104.24 (14)	C12—C11—C10	118.79 (15)
C4—C3—C2	115.85 (15)	C12—C11—H11	120.6
C4—C3—H3	122.1	C10—C11—H11	120.6
C2—C3—H3	122.1	C11—C12—C13	121.22 (16)
F1—C4—C3	117.79 (16)	C11—C12—H12	119.4
F1—C4—C5	117.77 (16)	C13—C12—H12	119.4
C3—C4—C5	124.43 (18)	C14—C13—C12	118.25 (16)
C6—C5—C4	119.70 (16)	C14—C13—H13	120.9
C6—C5—H5	120.2	C12—C13—H13	120.9
C4—C5—H5	120.2	F2—C14—C13	117.58 (16)
C5—C6—C7	116.84 (16)	F2—C14—C15	118.89 (16)
C5—C6—H6	121.6	C13—C14—C15	123.53 (15)
C7—C6—H6	121.6	C14—C15—C10	116.82 (15)
O1—C7—C6	126.08 (15)	C14—C15—H15	121.6
O1—C7—C2	110.41 (14)	C10—C15—H15	121.6
C6—C7—C2	123.51 (17)		

### Hydrogen-bond geometry (Å, º)

**Table:**

*D*—H···*A*	*D*—H	H···*A*	*D*···*A*	*D*—H···*A*
C5—H5···F2^i^	0.93	2.45	3.302 (2)	153
C15—H15···O3^ii^	0.93	2.47	3.3754 (19)	165

Symmetry codes: (i) *x*−1/2, −*y*+1/2, *z*−1/2; (ii) −*x*+1/2, *y*−1/2, −*z*+3/2.
